# Efficacy and safety of tetramethylpyrazine phosphate on pulmonary hypertension: study protocol for a randomized controlled study

**DOI:** 10.1186/s13063-019-3770-0

**Published:** 2019-12-16

**Authors:** Yuqin Chen, Wenjun He, Haiping Ouyang, Chunli Liu, Cheng Hong, Tao Wang, Kai Yang, Wenju Lu, Jian Wang

**Affiliations:** 1 State Key Laboratory of Respiratory Disease, National Clinical Research Center for Respiratory Disease, Guangzhou Institute of Respiratory Health, The First Affiliated Hospital of Guangzhou Medical University, 151 Yanjiang Road, Guangzhou, Guangdong 510120 People’s Republic of China; 2 Division of Translational and Regenerative Medicine, Department of Medicine, The University of Arizona College of Medicine, Tucson, AZ USA

**Keywords:** Pulmonary hypertension, Tetramethylpyrazine, Randomized controlled study

## Abstract

**Background:**

Tetramethylpyrazine (TMP), an active ingredient in the traditional Chinese herbal medicine Rhizoma Chuanxiong, has been used clinically for the prevention and treatment of cardiovascular disease. The benefits of TMP are largely attributed to its anti-oxidative and vasodilative properties. However, the efficacy of TMP in the treatment of pulmonary hypertension (PH) is unknown. We hypothesized that TMP may have a therapeutic effect in patients with PH.

**Methods/design:**

A randomized, single-blinded, clinical study with a TMP treatment group and a control group will be conducted to evaluate the efficacy and safety of TMP intervention in patients with PH. The recruitment target is 120 subjects meeting the following criteria: (i) at rest and at sea level, mean pulmonary artery pressure above 20 mmHg and pulmonary capillary wedge pressure below 15 mmHg; (ii) type 1 or 4 PH in the stable phase; (iii) age 15–70 years; (iv) 6-min walk distance between 100 and 450 m; (v) World Health Organization (WHO) functional classification of pulmonary hypertension of II, III, or IV. Subjects will be assigned randomly into two groups at a ratio of 1:2 (control:TMP). Both groups will receive routine treatment, and the treatment group will also receive oral TMP (100 mg) three times a day for 16 weeks. All patients will be followed up for 4, 8, 12, and 16 weeks; symptoms and patient compliance will be recorded.

**Discussion:**

We aimed to determine the efficacy and safety of TMP for the treatment of PH.

**Trial registration:**

Chinese Clinical Trial Register, ChiCTR1800018664. Registered on 2 October 2018.

## Background

Pulmonary hypertension (PH) is a serious condition characterized by sustained elevated mean pulmonary arterial pressure over 20 mmHg and progressive right ventricle hypertrophy, leading to cardiac failure and even death [1–5]. With improvements in diagnostic techniques, PH is no longer a rare disease. According to the latest epidemiological data, the prevalence of PH is about 1% of the global population [6]. Understanding of the pathophysiology and pathogenesis of PH has increased and life quality of patients has improved significantly through the use of targeted drugs; however, combination therapy is more effective than mono-therapy, so it is important to find new drugs to improve efficacy [7–10]. In addition, the use of effective drugs is limited by their high cost, which largely restricts PH patients in developing countries accessing treatment because of the economic burden of these treatments. Therefore, it is imperative to develop new and affordable medications with strong efficacy and safety profiles.

Given its anti-oxidative, anti-myocardial injury, and vasodilative effects [11–14], tetramethylpyrazine (TMP), a traditional Chinese herbal medicine, is widely used in the treatment of cardiovascular and cerebrovascular diseases [15–18]. The pathogenesis of PH involves oxidative stress, vascular inflammation, and imbalance of intracellular calcium homeostasis [19–21]. In our previous study, we showed in a rat model of PH that TMP intervention improves calcium imbalance in pulmonary artery smooth muscle cells (PASMCs) by modulating the expression of TRPC1, TRPC6, Kv1.5, and Kv2.1, potentially through reducing the intracellular free calcium concentration to inhibit the contraction and proliferation of PASMCs and the remodeling of distal small pulmonary arteries. However, it has not yet been reported whether TMP has a therapeutic effect on PH. We therefore designed a 16-week, randomized, single-blinded, clinically controlled study to examine the efficacy and safety of TMP phosphate for the treatment of PH.

## Methods/design

### Study design

**Fig. 1:**
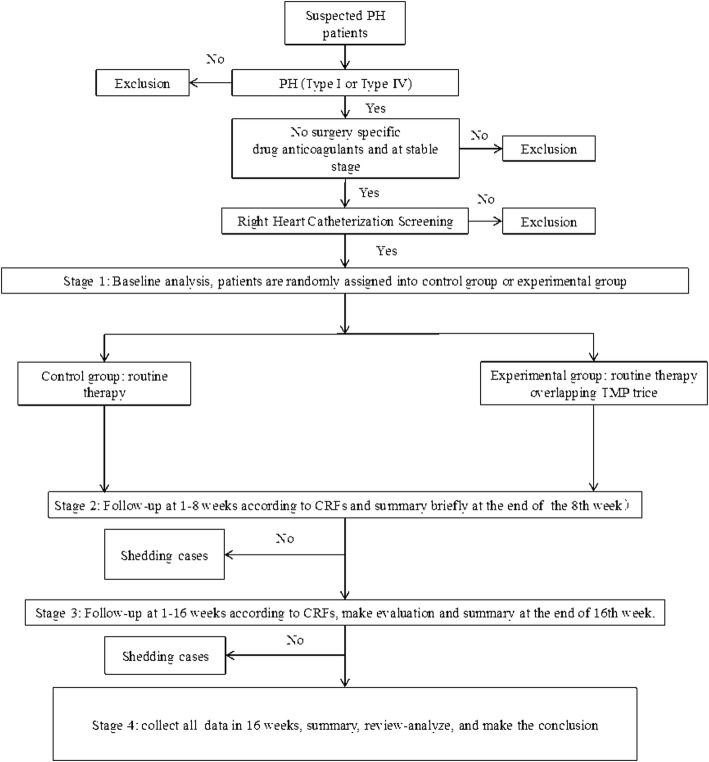
Flow chart for enrollment and follow-up of participants

We designed a protocol for a randomized controlled study. Screening (visit 0) is undertaken within 3 days prior to enrollment to assess eligibility and collect baseline data. Subjects who enter the primary screening are assessed for lung function, and those meeting all criteria will be randomly assigned (2:1) into a TMP treatment group or control group. Both groups will receive conventional treatment, but patients in the TMP treatment group will additionally receive 100 mg oral TMP (t.i.d). Patients will be followed up at week-4 after randomization (visit 1), and then followed up every 4 weeks until the end of treatment at 16 weeks. Data collected at visit 0 include patient characteristics (name, sex, age), medical history, concomitant medications, laboratory and auxiliary examinations, and adverse events. Additionally, at each visit, medical history, medications, cardiac and pulmonary function, and adverse events will be collected. Additional items will be evaluated at visits 2 and 4. A schedule of assessments is shown in Table 1. A study flow chart is shown in Fig. 1.

**Table 1:** Study schedule of assessments

Visit cycle evaluation projects	Screening stage	Visit 1	Visit 2	Visit 3	Visit 4
Time-Window	Day −3–0	Week 4	Week 8	Week 12	Week 16
Inclusion and exclusion criteria	√				
Informed consent	√				
Basic condition	√				
Basic medical history	√				
Complication	√	√	√	√	√
Symptoms and signs	√	√	√	√	√
Drug combination	√	√	√	√	√
Blood routine test, urine routine, liver and kidney function, coagulation function	√				√
Evaluation of cardiopulmonary function (6MWD, WHO-FC, Borg Score, MLHFQ)	√	√		√	√
Electrocardiogram	√				√
Imaging	√				
Arterial blood gases	√				√
NT-proBNP, cTNI levels	√				√
Echocardiography	√		√		√
Pulmonary function test	√		√		√
Adverse events		√	√	√	√

√, required

Basic medical history contains current medical history (symptoms and signs) and previous history

Evaluation of cardiac and pulmonary function includes a 6-min walk distance (6MWD), Minnesota Living with Heart Failure Questionnaire (MLHFQ), Borg Score, and World Health Organization functional class (WHO-FC)

Safety parameters include routine blood tests and urinalysis, liver and kidney function, and coagulation function

### Sampling

Based on the 6-min walk distance (6MWD) as one of the main efficacy indexes, it is assumed that, after treatment, the experimental group will be able to walk an average distance of 60 m further than the control group in 6 min, with standard deviation of 60 m, α of 0.05, and efficacy of 90% (β is 0.10). Thus, the required sample size is:

$$ \mathrm{N}=\frac{\left({\mathrm{q}}_1^{-1}+{\mathrm{q}}_2^{-1}\right){\left({\mathrm{t}}_{\raisebox{1ex}{$\upalpha $}\!\left/ \!\raisebox{-1ex}{$2$}\right.}+{\mathrm{t}}_{\upbeta}\right)}^2{\upsigma}^2}{\upsigma^2} $$

where q1 is the proportion of the experimental group and q2 is the proportion of the control group, q1 = 2/3, q2 = 1/3, N ≈ 107.

Assuming a dropout rate of 11%, the required sample size is approximately 120 participants, comprising 40 patients in the control group and 80 patients in the TMP group.

### Study procedure

#### Eligibility criteria for enrollment

The selection of participants will be based on the following inclusion and exclusion criteria.

##### Inclusion criteria

1. In accordance with the diagnostic criteria for PH, mean pulmonary arterial pressure measured by right cardiac catheterization above 20 mmHg and pulmonary capillary wedge pressure below 15 mmHg at sea level and pulmonary vascular resistance > 3 WU in a resting state.
2. Subjects with type 1 or type 4 PH classified according to the World Symposium on Pulmonary Hypertension [21] who are in a stable stage (under regular medications without fluctuation in one month), including idiopathic PH, hereditary PH, PH induced by drugs or toxins, PH associated with connective tissue diseases or congenital heart diseases (with no surgery/intervention within the previous 6 months) and chronic thromboembolic PH. For type 4 patients, surgical treatment is preferred for patients with surgical indications; patients with PH after surgery, patients without surgical indications, and nonoperable cases will undergo stabilization with anticoagulant drugs (such as warfarin) for at least 1 month prior to study participation.
3. Aged between 15 and 70 years, male or female.
4. WHO PAH functional classification II, IV, or V.
5. 6MWD of > 100 m and < 450 m at baseline.
6. Patients stable for at least 1 month after standard treatment, and patients who have not received treatment with interventional or surgical closure in the 6 months prior to participation.
7. Patient or his/her guardian agrees to participation of the patient in the study and provides written informed consent for participation.

##### Exclusion criteria

1. Absent or limited legal capacity
2. Pregnant or lactating women
3. Serious primary diseases in major organs
4. Mental or physical disability preventing the completion of 6MWD
5. Suspected or confirmed history of alcohol or substance abuse
6. Known allergy to the components of TMP
7. AST and ALT values more than three times the upper limit of normal, or creatinine clearance rate < 50 ml/min
8. Low systemic blood pressure (< 90/50 mmHg) or uncontrolled hypertension (blood pressure > 170/110 mmHg)
9. Prior use of the study drug and discontinuation or change in targeted drugs (e.g., endothelin receptor antagonists, phosphodiesterase type 5 inhibitors, and guanylate cyclase) in the 3 months prior to screening
10. Presence of an active infectious disease such as hepatitis A, hepatitis B, AIDS, tuberculosis, or connective tissue diseases
11. Presence of serious infection, especially pulmonary infections
12. Shock or other hemodynamically unstable conditions
13. Cirrhosis or portal hypertension caused by cirrhosis
14. Severe bleeding or bleeding tendency such as active peptic ulcer, intracranial hemorrhage, trauma, or other bleeding events
15. Acute or chronic organic diseases (except for dyspnea) or other conditions (such as limb diseases) that may result in the subject being unable to complete the study (especially the 6MWD)
16. Use or accidental use of foods or drugs that may impact test results during the treatment period (e.g., amirace, fenfluramine, dexfenfluramine, L-tryptophan, methamphetamine, and phenylflurazone)
17. Any other circumstances under which the investigator considers the patient to be unsuitable for participation in the study

#### Withdrawal criteria

1. Subjects having poor compliance with the dosing regimen
2. Use or accidental use of foods or drugs that may impact test results during the treatment period (e.g., amirace, fenfluramine, dexfenfluramine, L-tryptophan, methamphetamine, and phenylflurazone)
3. Subjects with incomplete key data that may affect the statistical analysis

#### Endpoint standards

1. Subjects experiencing serious adverse reactions leading to suspension or termination of treatment during the study
2. Subjects whose condition deteriorates during the study
3. Subjects who withdraw consent or are unable to complete the study because of other circumstances
4. Patients treated with targeted drugs for type 1 or 4 PH prior to testing who stopped treatment with the targeted drug for any reason during the study and did not reinitiate treatment
5. Death (from PH or another cause)

##### Interventions

Subjects satisfying all criteria will be assigned (2:1) randomly into two groups as follows:

1. TMP treatment group: TMP 100 mg three times daily in addition to routine therapy
2. Control group: routine therapy only

The routine therapy follows the 2015 ESC Guideline. The current treatment strategy for PAH patients can be divided into three main steps:

1. The initial approach includes general measures (physical activity and supervised rehabilitation, pregnancy, birth control and post-menopausal hormonal therapy, elective surgery, infection prevention, psychosocial support, adherence to treatments, genetic counseling and travel), supportive therapy (oral anticoagulants, diuretics, O_2_, digoxin), referral to expert centers and acute vasoreactivity testing for the indication of chronic Calcium Channel Blocker (CCB) therapy.
2. The second step includes initial therapy with high-dose CCB in vasoreactive patients or drugs approved for PAH in non-vasoreactive patients according to the prognostic risk of the patient and the grade of recommendation and level of evidence for each individual compound or combination of compounds.
3. The third part is related to the response to the initial treatment strategy; in case of an inadequate response, the role of combinations of approved drugs and lung transplantation are proposed.

It is important to monitor renal function and blood biochemistry in patients with diuretic use to avoid hypokalaemia and the effects of decreased intravascular volume leading to pre-renal failure.

Optimal medical treatment for Chronic Thromboembolic Pulmonary Hypertension (CTEPH) consists of anticoagulants and diuretics, and O_2_ in cases of heart failure or hypoxaemia.

TMP was produced by Livzon Pharmaceutical Group Inc. (Zhuhai, Guangdong Province, China), following the instructions of the People’s Republic of China Pharmacopoeia [22]. Routine therapy will not differ between the two groups and will include phosphodiesterase type 5 inhibitors (sildenafil and tadalafil). Where subjects have previously received targeted drugs for the treatment of PH, the regimen will remain unchanged.

#### Outcome measures

##### Efficacy indicators

The main efficacy indicators are 6MWD and heart rate recovery at 1 min after the 6MWD (Table 2).

**Table 2:** Efficacy indicators

Main efficacy indicators	Secondary efficacy measurements
6MWD HRR1	Pulmonary hypertension WHO classification Borg Dyspnea Score Minnesota Living with Heart Failure Questionnaire NT-proBNP cTNI RVSP Uric acid Volume of pericardial effusion Pulmonary artery diameter Diameter of the same layer of aorta Arterial oxygen saturation Time of clinical deterioration

Clinical deterioration is defined as the need to increase medication or change the therapeutic regimen for the treatment of PH, particularly inhaled, intravenous, or subcutaneous application of prostacyclin and its analogues; aggravated symptoms of right heart failure that do not respond to diuretics; atrial septostomy or death; lung transplantation; or hospitalization caused by exacerbation of PH

Other clinical symptoms and signs, biochemical indicators, and imaging indicators are recorded (Table 2) for comprehensive prognostic evaluation and risk assessment

Secondary efficacy measurements include the following 12 indicators (Table 2): PH WHO classification, Borg Dyspnea Score, Minnesota Living with Heart Failure Questionnaire, N-terminal pro-brain natriuretic peptide, cardiac troponin I, right ventricular systolic pressure evaluated by echocardiogram, uric acid, volume of pericardial effusion, pulmonary artery diameter assessed by CT, diameter of the same layer of aorta assessed by CT, arterial oxygen saturation, and time of clinical deterioration.

#### Safety evaluation

Symptoms and signs including respiration rate, heart rate, and blood pressure will be recorded at each visit. Laboratory tests will be performed within 3 days prior to enrollment and will include routine blood tests and urinalysis, liver function, renal function, coagulation function, NT-proBNP, and electrocardiography. Adverse events will be assessed and recorded in the case report form.

#### Evaluation of adverse events

Adverse events, including symptoms, signs, and physical or laboratory examination abnormalities, will be carefully evaluated. All adverse events must be judged for their character, severity, and potential relationship to the study treatment. The correlation between adverse events and study treatment is divided into five levels: definitely related, probabley related, possiblely related, possiblely unrelated, definitely unrelated.

##### Treatment allocation

As the study is single-blind, only the participant will be unaware of which treatment they receive; those responsible for their care and evaluation (treating team and research team) will know the allocation or coding of the treatment allocation. This blinding of the participant will be achieved by identical packaging and labeling of both the TMP tablet and matched placebo. Each container of TMP/placebo will be identified by a unique kit code. Randomized lists containing kit allocation will be computer-generated by the safety statistician and sent to the research investigator who will produce the kits and allocation sequence. The safety statistician will manage the kit codes in the kit logistics application, which is linked to the 24-h randomization system, and will maintain the back-up kit-code lists for each site.

### Data management and analysis

#### General considerations

Per protocol set will pick up from the full analysis set for analysis. Statistical analysis of the efficacy of the study will be performed using statistical data sets that meet the protocol. The data will be analyzed by two-sided *t* test, with categorical variables analyzed by χ^2^ test and rank variables by paired Wilcoxon rank sum test. The test level α is 0.05, and *P* values ≤ 0.05 will be considered statistically significant.

The methods that will be used to handle missing data are described for each analysis. As this is a single-blind study, the study statistician will be blinded to treatment group allocation throughout the study until the database has been locked and downloaded for final analysis. Only the safety statistician, supervising study statistician, back-up safety statistician, and authorized individuals will have access to the treatment group allocations prior to the final analysis.

#### Frequency of analyses

Outcome data will be analyzed once only, at the final analysis, although statistical monitoring of safety data will be conducted throughout the study and reported at agreed intervals. Final analysis will take place 16 weeks after the last patient is randomized.

#### Endpoint analysis

All analyses will be conducted using data from the intention-to-treat population, defined as all patients who undergo randomization regardless of non-compliance with the intervention. The primary endpoint will be analyzed in the per-protocol population to determine whether the results are sensitive to the exclusion of patients who violated the protocol (e.g., those patients who underwent randomization but were subsequently found to be ineligible). Primary and secondary analyses will be performed by an investigator who is unblinded to treatment allocation. Outcome measures will be analyzed by χ2 test and rank variables by paired Wilcoxon rank sum test appropriate for the data type. Such analyses will be adjusted for randomization minimization factors such as baseline values where applicable (such as age and sex). Baseline characteristics will be summarized for each randomized group.

#### Safety analyses

All patients who receive at least one dose of trial treatment will be included in the safety analysis set. The number of patients reporting a serious adverse event (up to 28 days after the last dose of treatment) and the details of all serious adverse events will be reported for each treatment group. The number of patients withdrawing from treatment will be summarized by treatment arm, along with the reasons for withdrawal. All safety analyses performed prior to final analysis will be undertaken by the safety statistician.

#### Subgroup analyses

No subgroup analyses are planned.

#### Adverse events

An adverse event is defined as any untoward medical occurrence (including deterioration of a pre-existing medical condition) in a patient who has been administered a medicinal product; the event does not necessarily have a causal relationship with this product. The occurrence of adverse events will be recorded at visits 1–4. At each visit, the research nurse will complete the adverse event checklist to determine whether the patient has experienced any of the expected adverse events. Only the occurrence and corresponding severity of adverse events will be recorded.

### Ethics

The present study is being conducted in accordance with the Declaration of Helsinki and relevant clinical study research regulations in China. The protocol was approved by the Ethics Committee of the First Affiliated Hospital of Guangzhou Medical University. Prior to participation, all subjects must provide written informed consent. Our protocol followed the SPIRIT 2013 Checklist, which provided specific details in the Additional file 1.

## Discussion

To date, many targeted drugs have emerged for the treatment of PH; however, these drugs are limited by unsatisfactory long-term efficacy and their expensive price. Ligustrazine is an alkaloid extracted from the traditional Chinese medicine herb *Ligusticum wallichiii* Franch (i.e., *Ligusticum chuanxiong* Hort). TMP has a variety of cardio-cerebral vascular pharmacological effects such as protection of vascular endothelium and antiplatelet, anti-ischemia reperfusion injury, antioxidative stress, etc. actions [22]. Moreover, TMP has been widely used in Chinese clinics for the treatment of occlusive cardiovascular and cerebrovascular diseases such as coronary heart disease, cerebral thrombosis, and vasculitis [22]. TMP has also been reported for the treatment of various diseases such as cor pulmonale, heart disease, kidney disease, portal hypertension, type 2 diabetes, tumors, and restenosis after coronary stenting in recent years [23–29]. At present, TMP phosphate tablets are one of the most commonly used drugs in clinical practice in China. Some basic research has shown that treatment with TMP decreases the permeability of the hypoxia-induced rat pulmonary microvascular endothelial cell monolayer such that it can be used to treat pulmonary hypertension [30, 31]. However, there is little evidence to date on the efficacy and safety of TMP therapy for PH. We conducted a literature search on the side effects of TMP; all the current reported side effects are from injections, including phlebitis, chills, fever, rash, itching, chest tightness, palpitations, dizziness, dyspnea, sore throat, and so on [32, 33]. We found that no report has yet evaluated the adverse events associated with oral TMP treatment, and there is also no information on the National Center for ADR, China website (http://www.cdr-adr.org.cn/). The clinical observation of our own hospital has confirmed that oral intake of TMP is safe, without obvious adverse reactions. Thus, the present clinical study is expected to provide evidence for the safety and efficacy of TMP, a new affordable potential treatment for PH.

There are some limitations in this clinical study. Due to TMP’s odor, we set the study as a single-blinded clinical study. Also, the scale of this clinical study is relatively small. We plan to conduct a subsequent large-scale clinical study comprehensively evaluating the efficacy and safety of TMP in the treatment of PH based on the findings of the present study.

## Trial status

Recruitment started in September 2018 and is planned to end in October 2018, with 120 patients randomized. Treatment with TMP finished in October 2019. We disposal the data at present. The current protocol version is 2.0, dated 28 September 2018.

## Additional file

**Additional file 1.** SPIRIT 2013 Checklist: Recommended items to address in a clinical trial protocol and related documents*.

## Data Availability

Not applicable.

## Abbreviations

6MWD: 6-min walk distance
PH: Pulmonary hypertension
TMP: Tetramethylpyrazine
WHO: World Health Organization
