# Correction to: Efficacy and safety of tetramethylpyrazine phosphate on pulmonary hypertension: study protocol for a randomized controlled study

**DOI:** 10.1186/s13063-020-04245-x

**Published:** 2020-03-30

**Authors:** Yuqin Chen, Wenjun He, Haiping Ouyang, Chunli Liu, Cheng Hong, Tao Wang, Kai Yang, Wenju Lu, Jian Wang

**Affiliations:** 1 State Key Laboratory of Respiratory Disease, National Clinical Research Center for Respiratory Disease, Guangzhou Institute of Respiratory Health, The First Affiliated Hospital of Guangzhou Medical University, 151 Yanjiang Road, Guangzhou, Guangdong 510120 People’s Republic of China; 2 Division of Translational and Regenerative Medicine, Department of Medicine, The University of Arizona College of Medicine, Tucson, AZ USA

**Correction to: Trials (2019) 20:725**

**https://doi.org/10.1186/s13063-019-3770-0**

After publication of our article [1] the authors have notified us of two typos in the Trial status.

- Originally published phrase

Recruitment started in September 2018 and is planned to end in October **2018**, with 120 patients randomized. Treatment with TMP finished in **October 2019**. We disposal the data at present. The current protocol version is 2.0, dated 28 September 2018.

- Correct phrase

Recruitment started in September 2018 and is planned to end in October **2019**, with 120 patients randomized. Treatment with TMP will be finished in **March 2020**. We disposal the data at present. The current protocol version is 2.0, dated 28 September 2018.
